# On the impact of mass screening for SARS-CoV-2 through self-testing in Greece

**DOI:** 10.3389/fpubh.2024.1352238

**Published:** 2024-03-06

**Authors:** Samuel Gilmour, Spyros Sapounas, Kimon Drakopoulos, Patrick Jaillet, Gkikas Magiorkinis, Nikolaos Trichakis

**Affiliations:** ^1^Operations Research Center, Massachusetts Institute of Technology, Cambridge, MA, United States; ^2^National Public Health Organisation, Athens, Greece; ^3^Department of Data Sciences and Operations, Marshall School of Business, University of Southern California, Los Angeles, CA, United States; ^4^Department of Hygiene, Epidemiology and Medical Statistics, School of Medicine, National and Kapodistrian University of Athens, Athens, Greece

**Keywords:** non-pharmaceutical interventions, epidemic modelling, COVID-19 testing, mass screening, compartmental models

## Abstract

**Background:**

Screening programs that pre-emptively and routinely test population groups for disease at a massive scale were first implemented during the COVID-19 pandemic in a handful of countries. One of these countries was Greece, which implemented a mass self-testing program during 2021. In contrast to most other non-pharmaceutical interventions (NPIs), mass self-testing programs are particularly attractive for their relatively small financial and social burden, and it is therefore important to understand their effectiveness to inform policy makers and public health officials responding to future pandemics. This study aimed to estimate the number of deaths and hospitalizations averted by the program implemented in Greece and evaluate the impact of several operational decisions.

**Methods:**

Granular data from the mass self-testing program deployed by the Greek government between April and December 2021 were obtained. The data were used to fit a novel compartmental model that was developed to describe the dynamics of the COVID-19 pandemic in Greece in the presence of self-testing. The fitted model provided estimates on the effectiveness of the program in averting deaths and hospitalizations. Sensitivity analyses were used to evaluate the impact of operational decisions, including the scale of the program, targeting of sub-populations, and sensitivity (i.e., true positive rate) of tests.

**Results:**

Conservative estimates show that the program reduced the reproduction number by 4%, hospitalizations by 25%, and deaths by 20%, translating into approximately 20,000 averted hospitalizations and 2,000 averted deaths in Greece between April and December 2021.

**Conclusion:**

Mass self-testing programs are efficient NPIs with minimal social and financial burden; therefore, they are invaluable tools to be considered in pandemic preparedness and response.

## 1 Introduction

The global emergence of the SARS-CoV-2 pathogen caught the entire world off guard. In 2020 and 2021, widespread community transmission of the pathogen during the COVID-19 pandemic led to unprecedented demand for healthcare resources that stretched national health systems beyond their capacity. Before effective vaccines for COVID-19 arrived at scale, public authorities used non-pharmaceutical interventions (NPIs) as their primary tools for slowing the transmission of SARS-CoV-2 and managing the stress placed on their health systems (1, 2). Of course, policymakers must carefully assess the expected positive and negative consequences of any NPI before implementing it, and studies that quantify these effects are therefore essential to improving global preparedness for future pandemics. Social distancing, hygiene measures, masking measures, and testing policies are examples of NPIs with clear evidence supporting their effectiveness in slowing the spread of SARS-CoV-2 (3–6) while also being cost-effective (7).

In this paper, we focus on mass screening, which is a testing policy where large groups of susceptible individuals (including those who are asymptomatic) take tests to detect and isolate infections before the disease can be transmitted (8). Mass screening has been shown to slow the spread of SARS-CoV-2 by simulation studies (6), as well as in retrospective studies of programs in Slovakia, Liverpool (United Kingdom), and South Tyrol (Italy) (9–11). Furthermore, whereas NPIs such as lockdowns are associated with tremendous explicit and implicit negative consequences (12, 13), including financial costs, disrupted education, and adverse effects on mental health, mass screening is particularly attractive for its limited negative consequences. These are primarily direct financial costs associated with procuring, distributing, and administering tests, which can reasonably be estimated prior to implementation (14). However, this does not imply mass screening is affordable, especially when expensive polymerase chain reaction (PCR) tests are used in large quantities (15).

The financial burden of mass screening can be reduced by using rapid antigen tests (RATs) in place of or alongside PCR tests (16). RATs are inexpensive compared to PCR tests, and because they are simple to administer at home as “self-tests”, they can be distributed to individuals on a much larger scale. From an epidemiological perspective, the trade-off associated with RATs is as follows. On one hand, tests can be taken frequently, and results returned in less than 30 min (17), meaning that detected infections can be isolated up to 48 h faster than when using PCR tests. On the other hand, RATs have lower sensitivity than PCR tests, returning more false negatives. Simulation studies suggest that testing frequency is more important than test sensitivity in containing an epidemic (18), but there are additional concerns over adherence to self-testing programs. Controlled studies have shown adherence rates generally exceed 70% (19–21), but these studies do not account for situations where citizens may lose their income by reporting a positive result and being disqualified from working. In any case, as of 2022, WHO has provided a “strong recommendation” for the use of self-testing programs to slow the spread of SARS-CoV-2 (22).

Though self-testing programs based on RATs were widely implemented during the COVID-19 pandemic (21), to date, no data-driven studies have examined the effect of a real-world program. This paper aims to address the deficit using detailed data from a program implemented in Greece in April 2021. The program covered a large fraction of the population by targeting several groups: students, teaching staff, civil servants, and private sector employees. For the most part, individuals within these groups were required by law to take two self-tests per week, regardless of symptoms of disease, and report the results through a centralized online platform. Outside these groups, the entire population was also encouraged to test, and free self-testing kits were occasionally distributed indiscriminately at scale—particularly preceding or following holidays that involved large gatherings of people.

Our work sought to quantify the effectiveness of the Greek self-testing program in curbing the spread of SARS-CoV-2, and in particular, its impact on averting hospitalizations and deaths. We also sought to obtain insights on best practices and lessons learned from an operational perspective. The analysis should serve as a reference point for policymakers who contemplate rolling out mass screening programs during future pandemics.

## 2 Materials and methods

The study aimed to quantify the impact of the Greek self-testing program on curbing the COVID-19 pandemic in Greece between April 4, 2021 (the date the program launched), and December 15, 2021 (the final date in our dataset). During this period a total of 60 million self-testing kits were distributed, and at the peak of the program, as many as 20% of the population took two self-tests within a single week.

A novel compartmental model was developed that tracked the evolution of the pandemic in Greece. The model is reminiscent of an SIR model but with important modifications that enabled relevant dynamics, such as self-testing, testing at healthcare providers (henceforth “regular testing”), and vaccination programs, to be modelled. Our model follows a similar structure to other compartmental models that have been proposed specifically to describe the asymptomatic infection caused by COVID-19 (23, 24), which has been estimated to make up 35% of all infections (25).

**Figure 1:**
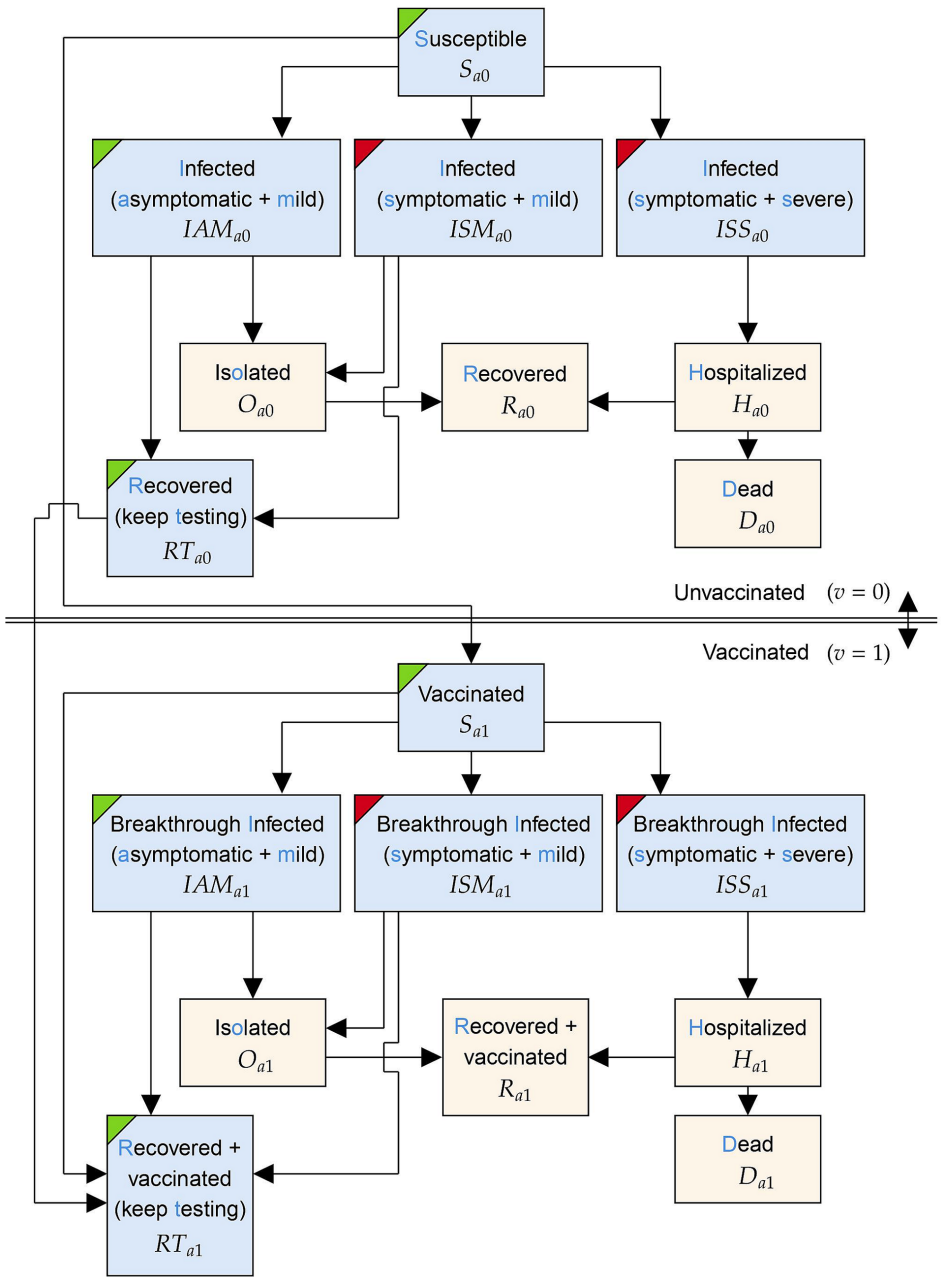
The structure of the model within a particular age group, *a*∈*G*. For some vaccination group *v*∈*{*0, 1*}*, the states within this age group are indexed by subscript *{av}*. Compartments coloured with a blue background are subject to testing. Compartments with green and red corners contain asymptomatic and symptomatic individuals, respectively.

At a high level, the initial compartments capture susceptible individuals who may become infected and contagious after being exposed to the virus. Infected individuals can be asymptomatic or symptomatic before either recovering with immunity or dying. Infected individuals may also be identified through testing, in which case they are isolated to avoid disease transmission. To capture the effects of the self-testing program, which were broadly allocated in different proportions amongst the age groups of 0 to 18 year-olds, 19 to 64 year-olds, and 65-plus year-olds, three sets of compartments corresponding to these age groups were considered. Depending on the age group, compartments are indexed with a subscript a taking values in $G=\left\{0-18,19-64,65+\right\}$ . Similarly, two sets of compartments were considered based on vaccination status, indexed by $v\in \left\{0,1\right\}$ to indicate vaccination. Figure 1 details the model compartments and possible transitions between them for a single age group, a∈G .

The model was fitted to highly detailed historical data obtained from the Greek National Public Health Organization (NPHO); a detailed explanation of the model, the data, fitting procedure, and other associated methods are provided in the Supplementary materials (Section B). The broad questions we sought to answer as part of the analysis were twofold:

1. What was the overall impact of the program in curbing the COVID-19 pandemic?
2. What can be learnt from the operational decisions made when implementing the self-testing program in Greece?

### 2.1 Overall impact of the program

To assess the overall impact of the self-testing program, its effect on three important metrics was estimated: (1) the reproduction number of the virus, (2) hospitalizations, and (3) deaths. These were estimated using two approaches, referred to as the direct and indirect methods. The direct method was used to analyse the reproduction number (calculated according to the methodology by Arroyo-Marioli et al. (26)), and both methods were used to analyse hospitalizations and deaths.

At a high level, the methods operated as follows. For the direct method, the model was fitted to the data, and then the total numbers of self-tests distributed across the age groups were set to zero. After this modification, with all other parameters held fixed at their fitted values, the model was used to simulate trajectories that the pandemic in Greece would have followed had the self-testing program not been implemented. A rigorous presentation of this approach is provided in the Supplementary materials (Section B.5).

Whereas the direct method perturbed the fitted model by setting the number of self-tests distributed to 0, the indirect method used only local perturbations of the fitted model (±1% modifications in the number of self-tests) and arguments from convex analysis to provide a conservative estimate of the effect on deaths and hospitalizations. Details of this method are again deferred to the Supplementary materials (Section B.5).

### 2.2 Impact of operational decisions

Subsequently the model was used to provide insights on three key operational decisions and trade-offs underlying the design and implementation of mass screening programs:

1. Scale: How many tests should be used?

The trade-offs associated with scale are straightforward: more tests come at higher cost, while increasing the potential for early detection and isolation of infections. Across the period of the study, over 60 million self-testing kits were distributed in total and on average 2.1% of the population were tested daily. The analysis aimed to quantify the effect on hospitalizations and deaths had the total number of self-tests distributed been scaled up or down. The precise technical details of how these estimates were produced can be found in the Supplementary materials (Section B.4).

1. Target: Which subpopulations should be targeted?

Given a fixed number of tests to be deployed, it is important to allocate them appropriately amongst age groups in the population. Greece launched its self-testing program in April 2021 by providing two self-testing kits per week to all school students and staff. In May 2021, private sector employees and civil servants who were not vaccinated or previously infected were required by law to carry out two weekly self-tests. On average, 56.2% of the self-tests were allocated to the 0–18 age group, 43.3% were allocated to the 19–64 age group, and 0.5% were allocated to the 65+ age group. The model was used to evaluate all alternative distributions of self-tests amongst age groups and estimate the allocation strategy which would have resulted in the fewest hospitalizations and deaths.

1. Accuracy: How important is the clinical accuracy of the self-tests and regular tests used?

Since self-tests are naturally less accurate than regular tests, and since the administration of tests by a member of the public instead of a medical professional could have further diminished the self-tests credibility, the model was used to estimate the effect of different self-test sensitivities on hospitalizations and deaths. The model was also used to estimate the effect of different regular test sensitivities on hospitalizations and deaths, primarily for preparedness in future pandemics where diagnostic tests may not share the same characteristics as those for SARS-CoV-2.

## 3 Results

### 3.1 Overall impact of the program

Table 1 presents 80% confidence intervals for the average reduction and the maximum weekly reduction in the effective reproduction number, $R_{t}$ , over the period of study. The analysis suggests that the program reduced the transmissibility of the virus by 4.7% on average, with a largest weekly reduction of approximately 24%.

**Table 1:** Estimates for the percentage reduction in *R_t_*, and absolute reduction in deaths and hospitalizations due to the self-testing program in Greece (values in parentheses provide 80% confidence intervals).

Metric	Estimate
**Percentage reduction in** *R_t_*	
Mean	4.72 (3.93–5.37)
Maximum	24 (17.3–25.6)
**Reduction in deaths**	
Using direct method	4,888 (3,698 – 6,808)
Using indirect method	3,434 (2,350 – 4,387)
**Reduction in hospitalizations**	
Using direct method	40,655 (33,625 – 50,699)
Using indirect method	28,379 (21,763 – 34,425)

Table 1 also presents 80% confidence intervals on the number of deaths and hospitalizations averted by the self-testing program, as obtained by the direct and indirect methods of estimation detailed in the Supplementary materials (Section B.5). For reference, the total number of deaths observed in the historical data over the self-testing period was 10,336, and the total number of hospitalizations was 76,299.

The most conservative estimates on the effect of the self-testing program suggest a mortality reduction of at least 20%, which corresponds to approximately 2,000 deaths. Furthermore, the program yielded a reduction in hospital admissions of at least 25%, corresponding to approximately 20,000 hospitalizations.

### 3.2 Impact of operational decisions

#### 3.2.1 Scale of program

**Figure 2:**
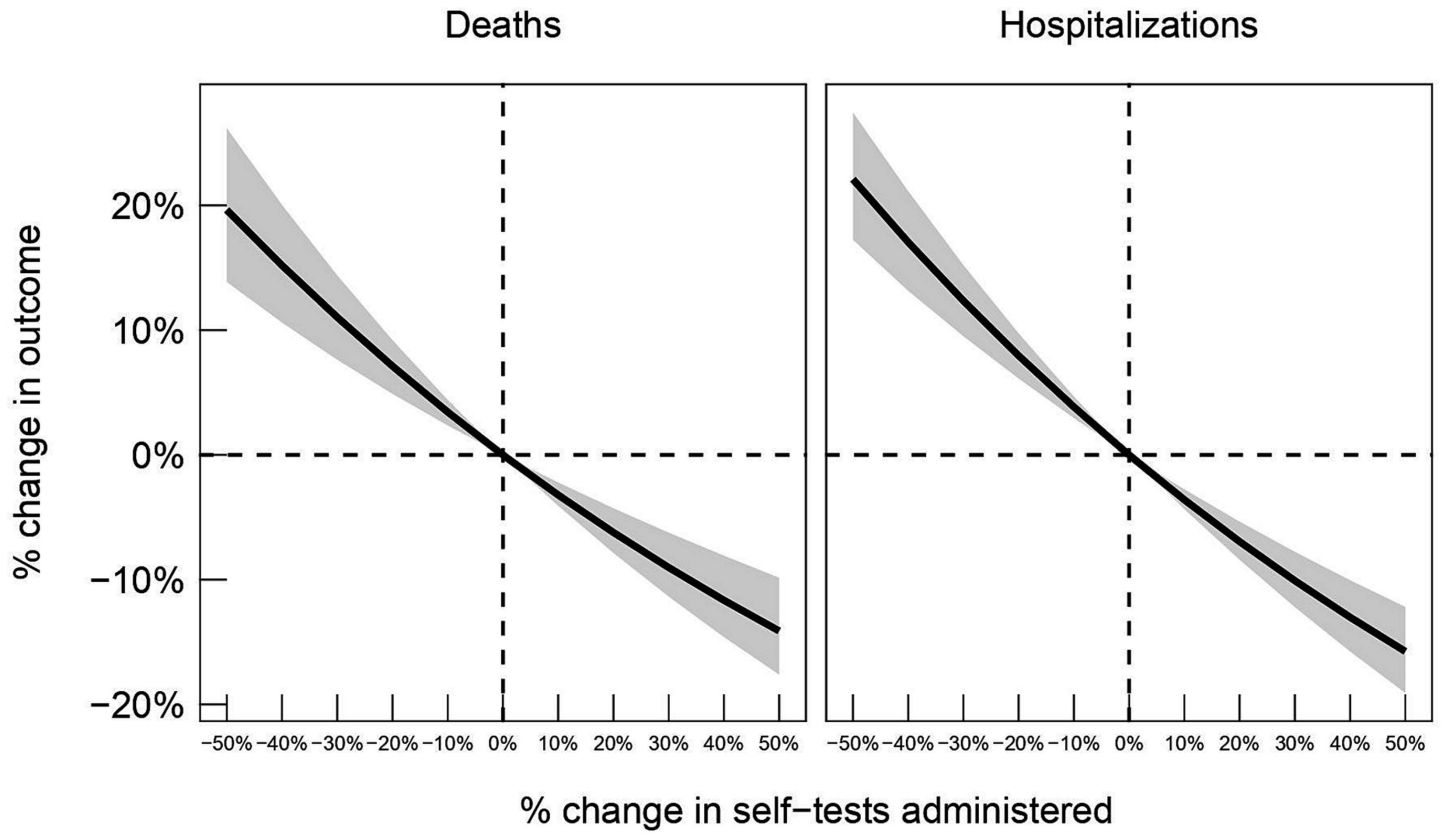
80% confidence intervals for the percent change in deaths (left panel) and hospitalizations (right panel) as a function of the percent change in the self-tests administered, relative to numbers observed in the data.

Figure 2 provides estimates of the percentage change in hospitalizations and deaths had the program been scaled up or down by some factor relative to the implementation in Greece. For example, if 20% fewer tests had been administered, total deaths would have increased by approximately 5% and total hospitalizations by approximately 8% during the period of study.

#### 3.2.2 Target population age groups

**Figure 3:**
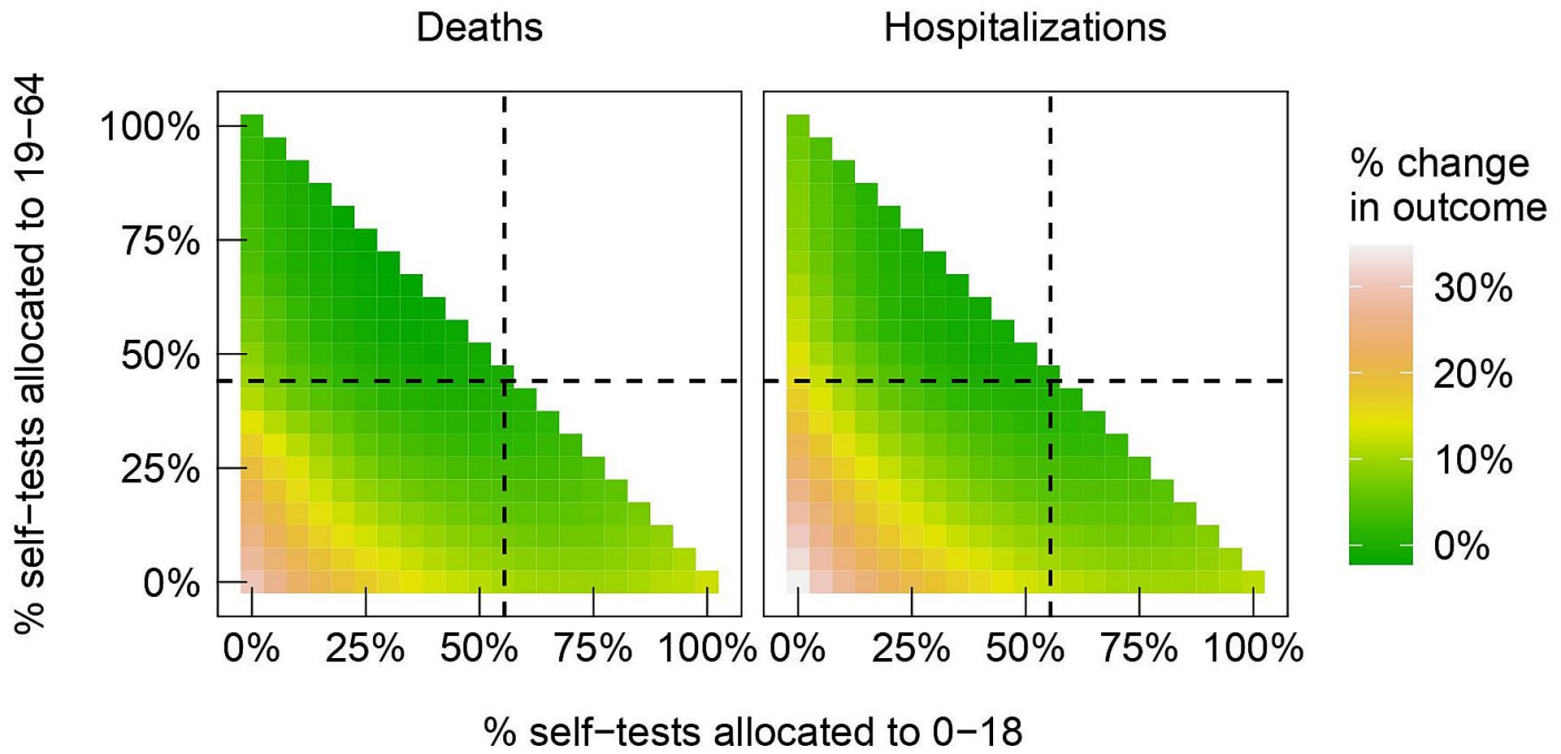
Heatmap of percent changes in deaths (left panel) and in hospitalizations (right panel), relative to what is observed in the data, as we vary the fractions of tests between different age groups: the 0–18 is allocated the percentage in the x-axis; the 19–64 age group is allocated the percentage in the y-axis; the 65+ group is allocated the remainder. Dashed lines indicate the fractions observed in the data.

Percentage reductions of deaths and hospitalizations for all possible distribution strategies of self-tests are provided in Figure 3, and the largest reductions are as follows. A strategy that distributed 30% of self-tests to the 0–18 age group and 70% to the 19–64 age group resulted in a 2.23% reduction in deaths (80% CI: −3.85 to 6.84%). A strategy that distributed 40% of self-tests to the 0–18 age group and 60% to the 19–64 age group would have resulted in a 1.16% reduction in hospitalizations (80% CI: −2.34 to 4.04%).

#### 3.2.3 Accuracy of tests

**Figure 4:**
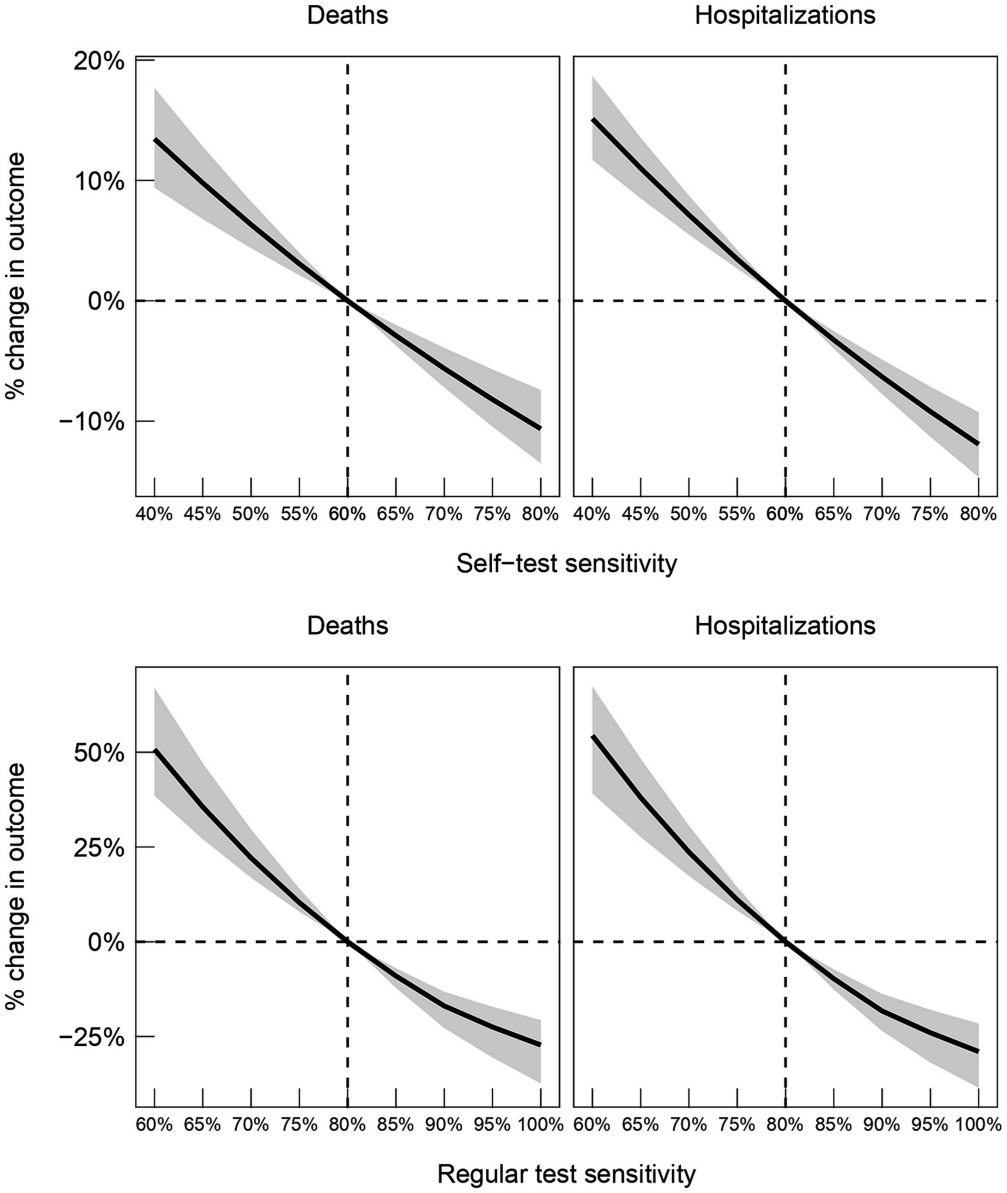
80% confidence intervals for percent changes in deaths (left panels) and hospitalizations (right panels), relative to what is observed in the data, as the sensitivities of self-tests and regular tests are varied. Dashed line indicates the sensitivities reported by the manufacturers of the tests used in the Greek testing program.

Figure 4 presents estimates on the percentage changes in averted deaths and hospitalizations, relative to what was observed in practice, for different values of the sensitivity of self-tests and regular tests. The results indicate that higher quality self-tests would have contributed to averting more deaths and hospitalizations. For example, if sensitivity of self-tests was increased from 60 to 80%, we estimate that approximately 10% more deaths and 12% more hospitalizations would have been averted. On the other hand, we estimate that a reduction in sensitivity from 60 to 40% would have led to approximately 13% more deaths and 16% more hospitalizations. For regular tests, we estimate that an increase in sensitivity from 80 to 100% would have led to approximately 30% fewer deaths and hospitalizations, while a reduction in sensitivity from 80 to 60% would have led to approximately 50% more deaths and 55% more hospitalizations.

## 4 Discussion

NPIs based on social distancing, such as bans on public events, school closures, and full lockdowns, have been shown to reduce transmissibility of a pathogen (27), but are associated with large financial and societal costs. In contrast, self-testing for mass screening is a low-cost solution with minimal impact on social and economic activity—but the effectiveness of a real-world self-testing program in response to a widespread infectious disease has not been directly studied in the past. Our results suggest that the implementation of the self-testing program in Greece during 2021 was at least as effective in reducing the transmissibility of SARS-CoV-2 as the previously mentioned social distancing measures, and averted a significant number of hospitalizations and deaths.

Our results also show the scale of the self-testing program in Greece (as measured by the number of tests) yielded diminishing returns on deaths and hospitalizations avoided. On the one hand, more deaths and hospitalizations would have been averted had the program been scaled up and more tests been administered; on the other hand, disproportionally more deaths and hospitalizations would have occurred had the program been scaled down and fewer tests administered. The analysis also shows that alternative allocations of self-tests amongst the age groups could have been more effective in Greece. In particular, increasing the fraction of tests distributed to the 19–64 age group could have averted slightly more deaths and hospitalizations, which is consistent with findings that this age group have showed higher transmissibility compared to 0–18 year-olds and 65-plus year-olds (28). Finally, the analysis shows that using tests with higher sensitivity would have averted more deaths and hospitalizations.

Our study is unique in that it specifically aims to quantify the effect of a self-testing program, but the results suggesting that the program reduced hospitalizations and deaths are consistent with other retrospective studies on general mass screening programs (though direct comparisons are not possible due to differences in the study settings). For example, a synthetic control study on the impact of mass community testing in Liverpool, UK over a two month period associated the program with a 43% reduction in hospitalizations (10). A study on a similar program in South Tyrol, Italy suggested it reduced COVID-19 cases by 51% over a 40 day period (11). These retrospective studies sit alongside several stylised models, in which it is easy to show that mass testing has a significant impact on cases (6, 29). Mass screening is ultimately an effective NPI, and our results support the notion that self-testing, with its reduced financial burden, is an effective tool for implementing such programs.

### 4.1 Limitations

Given that the analysis was based on observational data and not a (natural or designed) experiment, several assumptions were made in developing the model. First, it was assumed that the dynamics of the disease followed a structure defined by a compartmental model (presented in Section B of the Supplementary materials)—though structures of this kind are widely used in the mathematical epidemiology literature and similar models have been used to assess testing policies (23). Furthermore, when performing the sensitivity analysis, other changes related to population behavior, policy modifications, or additional stress on the healthcare system caused by the perturbation were not accounted for (besides the potential test substitution effect discussed in Section B.4 of the Supplementary materials).

Moreover, the model assumed that false positive self-tests in susceptible individuals do not lead to them being isolated or hospitalized, since in principle these cases were followed up with a PCR or antigen test and resolved. Another assumption was that individuals die only after being hospitalized, implying there are no deaths at home. The data fully supports this latter assumption—there were almost no COVID-19-related deaths outside of a hospital in Greece. Finally, although individuals age, these transitions between compartments of different age groups were not modelled for simplicity.

### 4.2 Implications for policymakers

Despite its shortcomings, the model offers insights for policymakers and public health practitioners seeking to deploy and optimise a self-testing program. First, our results suggest the Greek program was effective because it focused on frequent testing of a large proportion of the population, even though the tests themselves were of lower sensitivity. This offers support for the hypothesis developed following the mass screening program undertaken in Slovakia, where the reproduction number fell immediately after the testing program but quickly increased again when high-intensity testing was not sustained (30). This finding is also supported by simulation (18) and smaller scale studies (31), therefore setting an important priority for policymakers responding to future pandemics: frequent testing is critically important.

For policymakers operating in cost-or resource-constrained environments where frequent screening of the entire population is not feasible, our results suggest that self-testing should target subsets of the population with the highest transmissibility, which previous studies have suggested is the group of working age adults in the 19–64 bracket (28, 32). Furthermore, while self-tests with higher sensitivity should always be preferred due to their ability to detect more cases, our results suggest that an effective implementation can likely still be achieved with a reduced financial burden by using low sensitivity self-tests (provided the coverage and frequency of testing within the population is still sufficiently high). Our results also suggest that a self-testing campaign must be supported by regular tests (which are administered at healthcare providers) with high sensitivity—in other words, self-tests are not a substitute for regular tests.

A significant challenge for policymakers is ensuring its population has high rates of adherence in taking and reporting self-tests. Though adherence rates have generally exceeded 70% in controlled studies (19–21), actual rates depend on many factors such as trust in authorities, work-from-home patterns and social support. Policymakers must be prepared to develop strategies that address the sources of missing and misreporting tests. However, it should be noted that an adherence rate less than 100% effectively reduces the sensitivity of a self-test, and our analysis has shown a self-testing program can be effective even with imperfect adherence.

The success of the Greek self-testing program relied on some key operational successes which should not be taken for granted by other countries seeking to implement a similar program. Though self-testing kits have been shown to be stable to short fluctuations of temperatures outside their extremes (33), distributing nearly 70 million kits throughout Greece relied on a robust supply chain. Furthermore, Greece was quick to develop the online reporting platform which linked the self-reported result of each test to a social security number, and ultimately provided useful surveillance statistics. Policymakers seeking to implement a self-testing program should not overlook these important components of the program; WHO provides a comprehensive implementation guide that many relevant considerations (22).

The potential for future pandemics appears to be increasing as the global population grows and becomes more mobile and interconnected. It is important to document interventions that were used during the COVID-19 pandemic to develop a set of best practices for future pandemic preparedness. Mass screening through self-testing is one such best practice that could provide an invaluable tool to slow the spread of a highly infectious pathogen with minimal social and financial cost.

## Data availability statement

The majority of data used in the study is taken from daily Covid-19 reports released to the public by the National Public Health Organisation of Greece (NPHO). These reports provide information such as case numbers, hospitalizations, and deaths for different regions within the country. In the study, publicly-available information is supplemented with internal NPHO datasets that provide the self-testing numbers, as well as other detailed information such as daily vaccinations split by region and hospitalizations split by vaccination status. This internal data is anonymized and access can be provided upon request. Access to codes used to conduct all analyses is also available upon request.

## Ethics statement

Ethical approval was not required for the study involving humans in accordance with the local legislation and institutional requirements. Written informed consent to participate in this study was not required from the participants or the participants’ legal guardians/next of kin in accordance with the national legislation and the institutional requirements.

## Author contributions

SG: Formal analysis, Investigation, Methodology, Visualization, Writing – original draft, Writing – review & editing. SS: Formal analysis, Investigation, Methodology, Writing – original draft, Writing – review & editing. KD: Methodology, Writing – original draft, Writing – review & editing. PJ: Writing – review & editing. GM: Formal analysis, Investigation, Methodology, Writing – original draft, Writing – review & editing. NT: Formal analysis, Investigation, Methodology, Writing – original draft, Writing – review & editing.
