# Supplementary material for: On the impact of mass screening for SARS-CoV-2 through self-testing in Greece
Source: Front Public Health. 2024 Mar 6;12:1352238. doi: 10.3389/fpubh.2024.1352238 (PMC10950936; doi:10.3389/fpubh.2024.1352238)
Supplement: Supplementary file 1 [file Data_Sheet_1.PDF]

On the Impact of Mass Screening for SARS-CoV-2 through Self-Testing in Greece

Samuel Gilmour * Spyros Sapounas † Kimon Drakopoulos ‡ Patrick Jaillet *
Gkikas Magiorkinis § Nikolaos Trichakis *

February 13, 2024

Supplementary Materials

A. Additional Program Implementation Details

In March 2021, when a new wave of the COVID-19 pandemic placed significant pressure on the Greek National Health System, authorities chose to launch mandatory weekly testing, starting with students and staff in schools. Self-testing kits were distributed through pharmacies across the country on a weekly basis, using social security numbers. Control was carried out either with the display of a solemn declaration stating that the individual was tested negative, or through an online form available at `self-testing.gov.gr`. For positive self-test results, an amendment of legislation required a second test (either PCR or antigen) by a healthcare professional to confirm the result of the first test and update the National Registry for COVID-19 Patients. The authorities also proceeded occasionally with the distribution of free self-testing kits to the entire population, particularly before or after public holidays.

The distribution of self-testing kits and the implementation of mandatory checks began on April 7, 2021, on the reopening date of high schools, vocational high schools and junior high schools as well as junior high schools with senior classes, with the provision of two self-testing kits per week for all students (as well as the staff at these schools). On April 19, 2021, the government passed a law imposing mandatory self-testing for private sector employees and civil servants working physically

*Operations Research Center, Massachusetts Institute of Technology, Cambridge, MA 02139
†National Public Health Organisation, Athens 15123, Greece
‡Department of Data Sciences and Operations, Marshall School of Business, University of Southern California, Los Angeles, CA 90089
§Department of Hygiene, Epidemiology and Medical Statistics, School of Medicine, National and Kapodistrian University of Athens, Athens 15772, Greece

at their workplace. Self-testing kits were distributed to these employees throughout the summer period of 2021, while distribution to teaching staff and students paused during the summer holidays.

In September 2021, authorities imposed once-per-week mandatory self-testing for private sector employees and civil servants who were not fully vaccinated or who have not previously had COVID-19, at personal cost. With the beginning of the new school year, weekly self-testing was mandatory only for students aged 4-18 who were not vaccinated or had not previously had COVID-19. It should be noted that when students were considered as close contacts of a COVID-19 case, the Education Division would provide additional self-tests to ensure daily testing. Finally, private sector employees and civil servants who were not vaccinated or had not had COVID-19 were obliged to carry out two weekly tests from November 5, 2021, onwards, at personal cost, considering the role of seasonality in the spread of SARS-CoV-2.

During the entire period, a total of 97,000,000 self-testing kits were purchased. The kits were selected based on the rates of sensitivity (above 85% for samples testing positive with a qPCR up to the 33rd reaction cycle) and specificity (over 99%), which was supported through a peer reviewed process available at the FIND test directory (`https://www.finddx.org`). Importantly, these tests were safe, inexpensive, acceptable by the target population and simple to take at home or anywhere, providing rapid results.

B. Model Description

This section describes the model and methods used for the analyses in detail. It begins with a qualitative description of the compartments and transitions and then elaborates on the model dynamics – in other words, how the transitions were modelled using a mix of parameters and available historical data. This is followed by a discussion of the methods used to fit the model (Section B.3), conduct general sensitivity analyses (Section B.4), and evaluate the impact of the Greek self-testing program (Section B.5). A detailed description of the procedure which minimized the loss function in order to fit the model is left to Section C.

B.1. Model Compartments and Transitions

Recall from the short description provided in the Materials and Methods that three sets of compartments based on age are considered, $\mathcal{G} = \{0-18,\ 19-64,\ 65+\}$, and two sets of compartments based on vaccination status, indexed by $v \in \{0, 1\}$ to indicate vaccination.

?? illustrated, for a single age group $a \in \mathcal{G}$, the model compartments and the possible transitions

between them. For the unvaccinated population, susceptible individuals (S_{a0}) may transition to become vaccinated (S_{a1}), or infected. If infected, they move to one of three compartments: asymptomatic and mild (IAM_{a0}), symptomatic and mild (ISM_{a0}), or symptomatic and severe (ISS_{a0}). Populations in all three of these compartments can spread the disease to other populations for which infection is possible.

Infected individuals may be identified by taking a test, at which point those with mild disease move to an isolated compartment (O_{a0}), whereas those with severe disease move to a hospitalizated (H_{a0}) compartment. Infected individuals with mild disease, however, may never be identified through testing – in which case they eventually recover into a compartment that continues to be subject to testing (RT_{a0}). Infected individuals with severe disease are always eventually identified.

Populations in isolation with mild disease eventually recover, while hospitalized populations may recover or die. Recovered individuals transition to R_{a0}, and those who die to D_{a0}.

For the vaccinated population, transitions between compartments follow the same structure as with the unvaccinated, but with the following exception: because vaccination could confer full immunity for a fraction of the vaccinated population, vaccinated individuals in S_{a1} may transition directly to the recovered compartment that continues to be subject to testing (RT_{a1}) after being exposed to the virus.

B.2. Model Dynamics and Data

Guided by available data (which was mostly derived from daily records), the model considers discrete time steps corresponding to days. Time steps are indexed with $t \in \{0, 1, \ldots, T\}$, where T is the model horizon.

The dynamics of the model can be described in terms of the number of individuals that transition between any two compartments in a single time step. To introduce some notation, let $\mathcal{X}$ be the set of all compartments and $\mathcal{X}_{av}$ the set of all compartments for a given age group a and vaccination status v. For compartments $X, Y \in \mathcal{X}$, the number of individuals that transition from X to Y at time step t is denoted $\Delta_{X \to Y}(t)$. For each compartment X with population at time step t equal to $X(t)$, the population at the next time step is given by:

$$X(t+1) = X(t) + \sum_{Y \in \mathcal{X}} \Delta_{Y \to X}(t) - \sum_{Y \in \mathcal{X}} \Delta_{X \to Y}(t).$$

Transitions for which historical data exists are modelled explicitly using the data, while other transitions are modelled using parameters learnt during the fitting process. To disambiguate, the

following notational convention is used: quantities that were explicitly available in data are denoted with an overbar, whereas quantities that are learnt during the fitting process are denoted with no overbar. For example, $\bar{x}$ denotes a quantity that is explicitly provided in the data, whereas x is a quantity that has to be learned.

Transitions are discussed in four groups: (i) Vaccination, (ii) Infection, (iii) Testing and Isolation, and (iv) Recovery, Hospitalization and Death.

Vaccination

Vaccinated individuals are those who have received two doses of the vaccine. Let $\bar{v}_a(t)$ denote the recorded number of individuals in age group a who received their second dose on day t. These data are provided by Greek National Public Health Organization (NPHO) records.[1]

The available data do not specify whether individuals who were vaccinated at time t belonged in the susceptible (S_{a0}) or recovered compartment that is subject to testing (RT_{a0}), which is why a proportional split between the two is assumed, so that:

$$\Delta_{S_{a0}\to S_{a1}}(t) = \frac{S_{a0}}{S_{a0} + RT_{a0}}\bar{v}_a(t)$$

$$\Delta_{RT_{a0}\to RT_{a1}}(t) = \frac{RT_{a0}}{S_{a0} + RT_{a0}}\bar{v}_a(t)$$

Infection

Infected individuals, by coming into close contact with others, might expose susceptible or vaccinated individuals to the virus and infect them. To model these infection dynamics it is useful to quantify, first, the total number of newly infected people, and second, how many of these people transition into the different infection compartments in the model (depending on disease severity).

For now, focus on the transmission between two age groups, say $a, b \in \mathcal{G}$, at time t. Consider an infected individual from age group b and, to begin with, assume that all the population from age group a that they might come in contact with is unvaccinated and susceptible. Then, let $\beta_{ab}(t)$ be the number of people from age group a that the infected individual from age group b comes in close contact with and infects them. Note that these parameters, which are referred to as **mixing parameters**, capture, among other factors, the infectivity of the pathogen, the contagiousness of the infected individuals, and the contact patterns between different populations.

Eventually, at time step t, each infected individual from age group b need not infect as many as $\beta_{ab}(t)$ people from group a due to two reasons: (i) some of those from age group a might not be

susceptible, and (ii) they may have developed immunity by being vaccinated.

Now focus on the unvaccinated people from group a. To calculate how many among them will become infected due to an infected person from group b, the fraction of susceptible and unvaccinated among the population that the infected individual might come in contact with needs to be factored in. The latter population, referred to as the **community population**, is drawn from all compartments, except from the isolated, hospitalized and dead compartments. The collection of aforementioned compartments is denoted $\mathcal{C}_a$, with:

$$\mathcal{C}_a = \{S_{av},\, IAM_{av},\, ISM_{av},\, ISS_{av},\, RT_{av},\, R_{bv} \,:\, v \in \{0,1\}\}$$

and the size of the associated community population is denoted $C_a(t)$, with:

$$C_a(t) = \sum_{X \in \mathcal{C}_a} X(t).$$

Using this notation, each infected individual from age group b will infect $\beta_{ab}(t) \times S_{a0}(t)/C_a(t)$ individuals from age group a.

To calculate how many unvaccinated people from group a will eventually become infected in total, all infected populations from each age group must be considered. Let $I_b(t)$ be the number of infected individuals at time t from age group b, given by:

$$I_b(t) = \sum_{v \in \{0,1\}} (IAM_{av}(t) + ISM_{av}(t) + ISS_{av}(t))$$

Letting $NI_{av}(t)$ be the total number of newly infected people from group a with vaccination status v at time t, the quantity $NI_{a0}(t)$ can now be expressed as:

$$NI_{a0}(t) = \sum_{b \in \mathcal{G}} \beta_{ab}(t) \frac{S_{a0}(t)}{C_a(t)} I_b(t)$$

The breakthrough infection dynamics for the vaccinated people from group a are similar, with one difference: a fraction of vaccinated individuals who come into close contact with someone infected and who would have otherwise been subject to a breakthrough infection, may not be infected at all due to vaccine-induced immunity. A parameter termed the **probability of vaccine immunity**, $\bar{p}^{\text{v}-\text{imm}}$, can be introduced to model this effect. Therefore, a vaccinated individual who is exposed to the virus and would have been otherwise infected had they not been vaccinated, eventually does not get infected (cf. does get infected) with probability $\bar{p}^{\text{v}-\text{imm}}$ (cf. $1 - \bar{p}^{\text{v}-\text{imm}}$).

This probability is assumed to remain the same across age groups. Note that this probability relates to the effectiveness of the vaccine.

Vaccine effectiveness for the Omicron variant is estimated to be around 80%.[2] This value is adjusted to $\bar{p}^{\text{v-imm}} = 85\%$ to take into account the higher vaccine effectiveness against non-Omicron variants that were prevalent in Greece throughout 2021 (accounting for vaccine types and variant proportions in the population[3]).

Using the probability of vaccine immunity, $NI_{a1}(t)$ can now be expressed as:

$$NI_{a1}(t) = (1 - \bar{p}^{\text{v-imm}}) \sum_{b \in \mathcal{G}} \beta_{ab}(t) \frac{S_{a1}(t)}{C_a(t)} I_b(t).$$

Newly infected people will develop symptoms and disease with varying severity. The following two parameters are introduced to model this effect:

(i) **Probability of Asymptomatic Disease** Let $\bar{p}_a^{\text{asymp}}$ be the probability that an infected individual does not develop symptoms. This probability is allowed to depend on age, with:

$$\bar{p}_{\text{0-18}}^{\text{asymp}} = 0.6, \quad \bar{p}_{\text{19-64}}^{\text{asymp}} = 0.35, \quad \bar{p}_{\text{65+}}^{\text{asymp}} = 0.35,$$

based on estimates reported for unvaccinated infected individuals.[4] As there is no data to suggest that the proportion of asymptomatic infection is modified as a result of vaccination, the same probabilities are used for breakthrough infections as well.

(ii) **Probability of Severe Disease** Let p_{av}^{severe} be the probability that an infected individual who is symptomatic will also develop severe disease, as opposed to mild disease. Recall that those with severe disease eventually require hospitalization.

Estimates[5] for the probabilities of infected and unvaccinated individuals who are hospitalized (1% for 0 to 18, 7% for 19 to 64, and 30% for 65+) do not quite correspond to p_{av}^{severe} in our model, which is a probability conditional on symptomatic disease. Therefore, this probability is left as a free parameter to be learnt in the fitting process.

Putting all this together, transitions of the susceptible population into the infected compartments can be expressed as follows:

$$\begin{aligned}
\Delta_{S_{av} \to IAM_{av}}(t) &= \bar{p}_a^{\text{asymp}} && \times && NI_{av}(t), \\
\Delta_{S_{av} \to ISS_{av}}(t) &= (1 - \bar{p}_a^{\text{asymp}}) p_{av}^{\text{severe}} && \times && NI_{av}(t),
\end{aligned}$$

$$\Delta_{S_{av}\to ISM_{av}}(t) = (1-\bar{p}_a^{\text{asymp}})(1-p_{av}^{\text{severe}}) \times NI_{av}(t).$$

Finally, note that the fraction of vaccinated susceptible individuals who come into close contact with someone infected, but have developed vaccine-induced immunity as discussed previously, transition directly to the recovered compartment that continues to be subject to testing (RT_{a1}). This transition can now be expressed as:

$$\Delta_{S_{a1}\to RT_{a1}}(t) = \bar{p}^{\text{v-imm}} \sum_{b\in\mathcal{G}} \beta_{ab}(t)\frac{S_{a1}(t)}{C_a(t)} I_b(t)$$

Testing and Isolation

The model allows for two types of testing: regular tests and self tests. Both are conducted with the aim of isolating infected individuals and preventing them from spreading the disease or providing them with the appropriate care in the case of severe illness. Since they have different effects on the model dynamics, both test types are described separately.

Regular tests are more accurate than self-tests, with clinical sensitivity and specificity given by $\bar{\sigma}^{\text{reg}} = 0.8$ and $\bar{\mu}^{\text{reg}} = 1$ respectively. Available data[6] provide the total number of regular tests administered in the country. Greek census data[7] are used in conjunction with historical information to produce the number of regular tests performed daily by age group a and vaccination status v, denoted by $\bar{T}_{av}^{\text{reg}}(t)$, where tests are assumed to have been allocated proportional to the sizes of different age and vaccination groups.

The number of $\bar{T}_{av}^{\text{reg}}(t)$ tests are split among different compartments. Letting $T_X^{\text{reg}}(t)$ be the tests taken by the population in compartment $X \in \mathcal{X}$, this split is modelled as follows. First, because newly hospitalized individuals were routinely tested upon admission:

$$T_{ISS_{av}}^{\text{reg}}(t) = \Delta_{X\to H_{av}}(t)$$

The remaining $\bar{T}_{av}^{\text{reg}}(t) - T_{ISS_{av}}^{\text{reg}}(t)$ are assumed to be split as follows. Because regular tests were not required by the state, individuals in different compartments had different **propensities** to take them. θ_a and $1-\theta_a$ are introduced to denote the testing propensity of asymptomatic and symptomatic individuals respectively, from which the **total propensity** of age group a and vaccination group v, denoted Θ_{av}, can be expressed as:

$$\Theta_{av} = \theta_a(S_{av}(t) + RT_{av}(t) + IAM_{av}(t)) + (1-\theta_a)ISM_{av}(t)$$

Then, the $\bar{T}^{\text{reg}}_{av}(t) - T^{\text{reg}}_{ISS_{av}}(t)$ tests are split in proportion with the testing propensity and size of each compartment:

$$T^{\text{reg}}_X(t) = \begin{cases} \left(\frac{\theta_a X(t)}{\Theta_{av}}\right)\left(\bar{T}^{\text{reg}}_{av}(t) - T^{\text{reg}}_{ISS_{av}}(t)\right) \\ \qquad \text{if } X \in \{S_{av}, RT_{av}, IAM_{av}\} \\ \left(\frac{(1-\theta_a) X(t)}{\Theta_{av}}\right)\left(\bar{T}^{\text{reg}}_{av}(t) - T^{\text{reg}}_{ISS_{av}}(t)\right) \\ \qquad \text{if } X = ISM_{av} \\ 0 \qquad \text{otherwise} \end{cases}$$

The total number of positive regular tests from compartment X is therefore given by:

$$P^{\text{reg}}_X(t) = \begin{cases} \bar{\sigma}^{\text{reg}} T^{\text{reg}}_X(t) & \text{if } X \in \{IAM_{av}, ISM_{av}\} \\ \Delta_{X \to H_{av}}(t) & \text{if } X = ISS_{av} \\ 0 & \text{otherwise} \end{cases}$$

Self tests are in general less accurate than regular tests, with clinical sensitivity and specificity given by $\bar{\sigma}^{\text{self}} = 0.6$ and $\bar{\mu}^{\text{self}} = 1$ respectively.

Available data[6] provide the total number of self tests administered to a group (age group a, vaccination status v) for each week. These are assumed to be uniformly distributed throughout the corresponding week and $\bar{T}^{\text{self}}_{av}(t)$ is left to denote the number of self tests performed on day t.

Self tests are required by the state and therefore a uniform distribution amongst individuals within the eligible compartments can be assumed. Therefore, the total number of self-tests administered to a compartment X can be calculated as:

$$T^{\text{self}}_X(t) = \begin{cases} \frac{X(t)\bar{T}^{\text{self}}_{av}(t)}{S_{av}(t)+IAM_{av}(t)+ISM_{av}(t)+RT_{av}(t)} \\ \qquad \text{if } X \in \{S_{av}, IAM_{av}, ISM_{av}, RT_{av}\} \\ 0 \qquad \text{otherwise} \end{cases}$$

The total number of positive self-tests in each compartment is then given by:

$$P_X^{\text{self}}(t) = \begin{cases} \bar{\sigma}^{\text{self}} T_X^{\text{self}}(t) & \text{if } X \in \{IAM_{av}, ISM_{av}, ISS_{av}\}, \\ 0 & \text{otherwise} \end{cases}$$

All individuals who are identified as positive and are not suffering from a severe infection move to the isolated compartment:

$$\Delta_{X \to O_{av}}(t) = P_X^{reg}(t) + P_X^{self}(t), \text{ for } X \in \{IAM_{av}, ISM_{av}\}$$

Those with a severe infection and identified as positive are hospitalized, as discussed next.

Recovery, Hospitalization and Death

Infected individuals with mild disease spend an average of $\bar{\tau}^{\text{rec}} = 10$ days[8] in the infected compartments (IAM_{av} or ISM_{av}) before infectiousness subsides. Therefore, in the absence of a positive test, the following rates for each age group a and vaccination status v can be written:

$$\Delta_{IAM_{av} \to RT_{av}}(t) = \frac{1}{\bar{\tau}^{\text{rec}}} IAM_{av}(t)$$
$$\Delta_{ISM_{av} \to RT_{av}}(t) = \frac{1}{\bar{\tau}^{\text{rec}}} ISM_{av}(t)$$

Similarly, it is assumed without loss that isolated individuals also spend an average of $\bar{\tau}^{\text{rec}}$ days before recovery:

$$\Delta_{O_{av} \to R_{av}}(t) = \frac{1}{\bar{\tau}^{\text{rec}}} O_{av}(t)$$

Infected individuals with severe disease, are hospitalized following $\bar{\tau}^{\text{hosp}}$ days after infection:

$$\Delta_{ISS_{av} \to H_{av}}(t) = \frac{1}{\bar{\tau}^{\text{hosp}}} ISS_{av}(t)$$

Hospitalised individuals are discharged either due to death or recovery. The average length of stay in the hospital, denoted by $\bar{\tau}_{av}^{\text{los}}(t)$, varies with age, vaccination, and month and is provided by the Greek NPHO.[9] Among the discharged individuals, the fraction p_a^{die} of those who die could be in principle estimated by the raw data. Unfortunately, the raw estimate varies substantially and therefore, it is learnt through the fitting process. Combining the above, the following transitions

are derived:

$$\Delta_{H_{av} \to R_{av}}(t) = (1 - p_a^{\text{die}}) \frac{1}{\bar{\tau}_{av}^{\text{los}}(t)} H_{av}(t)$$

$$\Delta_{H_{av} \to D_{av}}(t) = p_a^{\text{die}} \frac{1}{\bar{\tau}_{av}^{\text{los}}(t)} H_{av}(t)$$

B.3. Model Fitting

The model was fitted against the following key outcomes, each of which has associated historical data: daily recorded hospitalizations, deaths, total cases, and cases reported through the self-testing program. Parameter values were selected to minimize the sum of squared log errors between the model predictions for these outcomes and the data. The following subsection details the key outcomes and data, while the minimization procedure is left for Section C.

Hospitalizations and Deaths The number of daily hospitalizations (by age group and vaccination status) and deaths (by age group) are available from data recorded by the NPHO of Greece.[10] The corresponding model predictions are calculated by:

$$\Delta_{ISS_{av} \to H_{av}}(t), \text{ and, } \sum_{v \in \{0,1\}} \Delta_{H_{av} \to D_{av}}(t)$$

Total Cases The number of daily total new cases by age group are available from data recorded by the Greek NPHO.[11] The corresponding model predictions are given by:

$$\sum_{v \in \{0,1\}} \sum_{X \in \mathcal{X}_{av}} P_X^{self}(t) + P_X^{reg}(t)$$

Cases Reported through the Self-Testing Program The number of daily new cases by age group from the self-testing program are available from data recorded by the Greek NPHO.[11] Notably, these cases were predominantly recorded following a positive self test. In addition, it is suspected that some of these cases were erroneously recorded through the program following a positive regular test, in the absence of a positive self test. To account for this possibility, let $\gamma_a(t)$ be the fraction of regular positive tests for age group a at time t that were reported through the self-test program. The model predictions of the number of cases reported through the self-testing program is equal

to:

$$\sum_{v\in\{0,1\}}\sum_{X\in\mathcal{X}_{av}} P_X^{self}(t) + \gamma_a(t)P_X^{reg}(t)$$

Note that $\gamma_a(t)$ is assumed to vary with age and over time to account for different types of reporting errors as the system processes varying amounts of self tests in the population.

The data to which the model is fitted, and expressions for the corresponding model predictions are summarized below:

(i) **Hospitalizations:** $\Delta_{ISS_{av}\to H_{av}}(t)$

(ii) **Deaths:** $\sum_{v\in\{0,1\}} \Delta_{H_{av}\to D_{av}}(t)$

(iii) **Total Cases:** $\sum_{v\in\{0,1\}}\sum_{X\in\mathcal{X}_{av}} P_X^{self}(t) + P_X^{reg}(t)$

(iv) **Cases through Self-Testing Program:** $\sum_{v\in\{0,1\}}\sum_{X\in\mathcal{X}_{av}} P_X^{self}(t) + \gamma(t)P_X^{reg}(t)$

B.4. Sensitivity Analyses

For each sensitivity analysis, the model is fitted to the data and then simulated for different values of the parameter of interest, keeping other parameters constant and equal to their fitted values. These simulations are performed within a bootstrapping process, so as to obtain not just point estimates, but confidence intervals too. The simulations are described below, and the bootstrapping process is detailed in C.3.

To estimate the impact of the number of self tests deployed, each simulation varies the number of self tests taken by scaling them uniformly across time and different population groups. That is, at time t, the number of self tests taken by population in age group a and vaccination status v is set to be $(1+\eta)\times \overline{T}_{av}^{\text{self}}(t)$, where η is a scaling parameter. $\eta = 0$ recovers the baseline scenario that was implemented in reality, while for $\eta < 0$ (cf. $\eta > 0$), scenarios are considered in which the scale of the program is decreased (cf. increased).

A self-testing program of a different scale would have likely induced a population testing behavior different than the one observed in the baseline scenario. If self-tests were reduced, for example, some individuals who self-tested in reality and could no longer test under this reduced-scale scenario might seek to take a regular test instead (particularly if they were symptomatic). Ignoring this potential for behavioral change could overstate the effect of self tests, especially when self tests are reduced, i.e. for $\eta < 0$. To alleviate this concern, the following modification is made to the simulation. First, under the baseline scenario and for individuals within a given community

compartment $X \in \mathcal{C}_a$, the fraction of them who sought a regular test among those who did not take a self test can be computed:

$$q_X(t) = \min\left(1,\ \frac{T_X^{\text{reg}}(t)}{X(t) - T_X^{\text{self}}(t)}\right) \tag{1}$$

The fraction $q_X(t)$ is the propensity of the population in X to take a regular test when they are unable to take a self test. It is assumed that this propensity remains the same regardless of the scale of the self-testing program. Let $\tilde{q}_X(t)$ be the propensities that are produced after fitting the model to the data. Then, in the simulations for the sensitivity analysis, the number of regular tests taken are modified to be:

$$\tilde{q}_X(t)\left(X(t) - T_{X;\eta}^{\text{self}}(t)\right) \tag{2}$$

where $T_{X;\eta}^{\text{self}}(t)$ is the number of self-tests in compartment X, after the total number of self-tests are scaled by $(1+\eta)$.

To estimate the impact of the self test allocation mix among different age groups, the fractions of total tests allocated to each group are varied – subject to the fractions summing to one, so that to total number of tests is held constant. For each set of fractions considered, the number of tests taken within each age group is scaled uniformly across all time periods.

Finally, for the sensitivity analysis on the clinical sensitivity of the self-tests, the fitted parameters are simply used to simulate the outputs of the model for various test sensitivity values, $\bar{\sigma}^{\text{self}}$.

B.5. Overall Impact of the Program

To evaluate the overall impact of the program on deaths and hospitalizations, two methodologies are used (both of which rely on a sensitivity analysis with respect to the number of self tests taken, described above). These are referred to as the **direct** and **indirect** methods:

1. **Direct method** The analysis is conducted simply by setting $\eta = -1$ (which corresponds to the scenario of no self tests) to obtain estimates for deaths and hospitalizations.

2. **Indirect method** First, the sensitivity analysis is used to estimate local derivatives at the baseline scenario for deaths and hospitalizations, which we denote with ∂D and ∂H. In particular, the analysis is run for $\eta = -0.01$ and $\eta = 0.01$, i.e., it considers $\pm 1\%$ perturbations of the program's scale, and then uses finite differences to estimate ∂D and ∂H. Given that the total number of deaths and hospitalization are concave in the number of self-tests

conducted, i.e., additional self-tests exhibit diminishing returns, conservative estimates of the total number of deaths and hospitalization are provided by:

$$(\#\text{total number of self tests}) \times \partial D$$
$$(\#\text{total number of self tests}) \times \partial H$$

Finally, to evaluate the overall impact of the program on transmissibility, the effective reproduction number R_t is calculated for each time step.[12] Then, applying the direct method (i.e. setting $\eta = -1$) allows estimates for R_t to be obtained in the scenario where no self-tests are conducted.

C. Loss Function Minimization

The model is fitted by finding the parameter values that minimize the sum of squared log errors between the data and the model predictions for the outcomes discussed in Section B.3.

To introduce some notation, consider an age group a and time period t. Let $\overline{\Delta H}_a(t)$ and $\overline{\Delta D}_a(t)$ be the corresponding number of new hospitalizations and deaths, respectively; let $\overline{P}_a^{\text{tot}}(t)$ be the corresponding total number of cases recorded; and let $\overline{P}_a^{\text{conf}}(t)$ be the corresponding number of cases reported through the self-testing program. The data and corresponding predictions are summarized in Table 1.

A non-convex optimization algorithm is used to minimize the sum of squared log errors between the model predictions and the target data. In order to avoid overfitting, a regularization term is included in the loss function.

C.1. Parameters and Initialization

Certain model parameters are constrained in order to facilitate the fitting process. These are discussed next and summarized in Table 2.

The probabilities of severe disease p_{av}^{severe}, though learned from the fitting process defined below, are constrained to lie within the following intervals:

$$0 \leq p_{\text{0-18},v}^{\text{severe}} \leq 0.02, \quad 0 \leq p_{\text{19-64},v}^{\text{severe}} \leq 0.14, \quad 0 \leq p_{\text{65+},v}^{\text{severe}} \leq 0.6$$

which have widths twice the size of estimated values.[5] Similarly, all p_a^{die} are learnt and constrained to lie in intervals $[0, 2q_a]$ where q_a represents the total deaths divided by the total hospitalizations in age group a in a given wave, obtained from Greek NPHO data.[10]

The mixing parameters, $\beta_{ab}(t)$, and the reporting parameters, $\gamma_a(t)$, are assumed to be constant in 4 and 6 week intervals respectively. These limits were empirically established with the goal of enabling tractability and avoiding overfitting.

The data includes all dates between January 21, 2021 and December 15, 2021. During this period, Greece experienced two epidemic waves, one lasting from January 21, 2021 until June 20, 2021, and the other from June 21, 2021 until December 15, 2021. The self-testing program was introduced in the middle of the first of these waves and continued through the second wave. The model is fitted to both these waves in separate runs. For the first wave, the sizes of all compartments are initialized at 0 with the following exceptions. The number of initially hospitalized patients are directly estimated from the raw data. The size of the susceptible compartments and infected compartments are allowed to be nonzero (and are learned through the fitting process) subject to the following constraints: (i) the prevalence is assumed to be less than 0.3% (an upper bound supported by Bastani et al.[13]), (ii) the sum of all compartments sums to the population data per age group provided in the Greek census.[7]

For the second wave, the sizes of all compartments are initialized at their levels learned from the first wave fitting process, with the exception of the infected compartments that are learned through the second wave's fitting process. Similar to the first wave, a constraint is imposed that the sum of all compartments sums to the population data per age group provided in the Greek census.[7] Note that fitting the sizes of the infected compartments for the second wave is necessary, because the first-wave fit for these quantities at the end of the first wave might not be credible owing to end-of-horizon effects.

C.2. Loss Function

The loss function used to fit the model is the sum of squared log-errors, across time steps, age groups, and waves for all outcomes presented in Table 1.

There are more than 100 parameters to fit and hence the loss function is complex with no analytical gradients, making the underlying optimization problem a difficult learning task with the potential risk of overfitting. In order to obtain sparse solutions, a regularization scheme is used on the time-varying parameters ($\beta_{ab}(t)$ and $\gamma_a(t)$) with a two-stage block-minimization technique to ease the optimization burden.

The regularization is a standard penalty on the absolute differences in successive values of the time-varying parameters. In particular, two regularization parameters are introduced, λ_1 and λ_2,

and the following component is added to the loss function:

$$\lambda_1 \sum_{a\in\mathcal{A}} \sum_{b\in\mathcal{A}} |\beta_{ab}(t+1) - \beta_{ab}(t)| + \lambda_2 \sum_{a\in\mathcal{A}} |\gamma_a(t+1) - \gamma_a(t)|$$

The block-minimization approach works in two steps:

1. Numerically minimize the regularized loss function with the cross-group mixing parameters fixed to zero ($\beta_{ab}(t) = 0$ for $a \neq b$), and obtain an initial estimate on the unknown parameters.

2. Numerically minimize the loss function by varying all mixing parameters, $\beta_{ab}(t)$, keeping the rest fixed to their values from Step 1.

Both fitting steps are completed using the Levenberg-Marquardt algorithm[14] with random restarts. The regularization parameters are fixed in the optimization and chosen by searching through a grid. All computational experiments are run on the SuperCloud infrastructure.[15]

C.3. Bootstrapping for Confidence Intervals

To derive confidence intervals for our sensitivity analyses, an approach is followed that is similar to traditional bootstrapping. It relies on the data being split according to the 74 regional units of Greece, and uses this split to construct "virtual" populations that are obtained by randomly sampling regional units (with replacement) until the total population of the collection is at least 2 million. It was observed that the time series within individual regional units with small populations suffered from high variance, and therefore the 2 million resident threshold was selected so that the time series of the virtual populations were relatively stable. Figure 1 shows the profiles of the 25 bootstrap samples.

In the analysis we set $N = 25$ for tractability, and choose to report 80% confidence intervals. Confidence intervals for the quantities of interest are derived by completing the fitting procedure and analysis on each of these datasets.

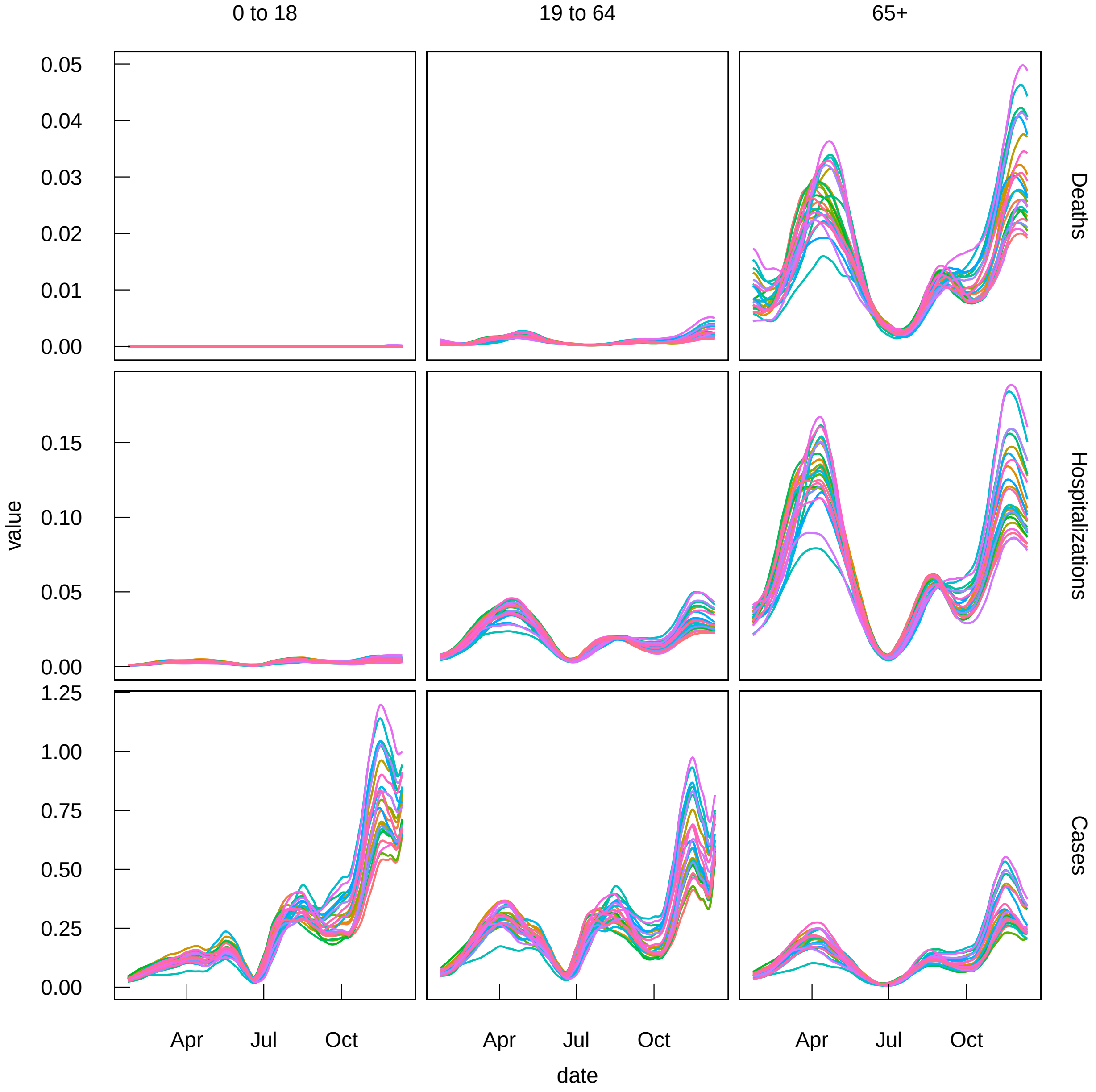

Figure 1: Deaths, hospitalizations, and cases per person split by age group. Each colored series corresponds to a time series from a single bootstrap sample.

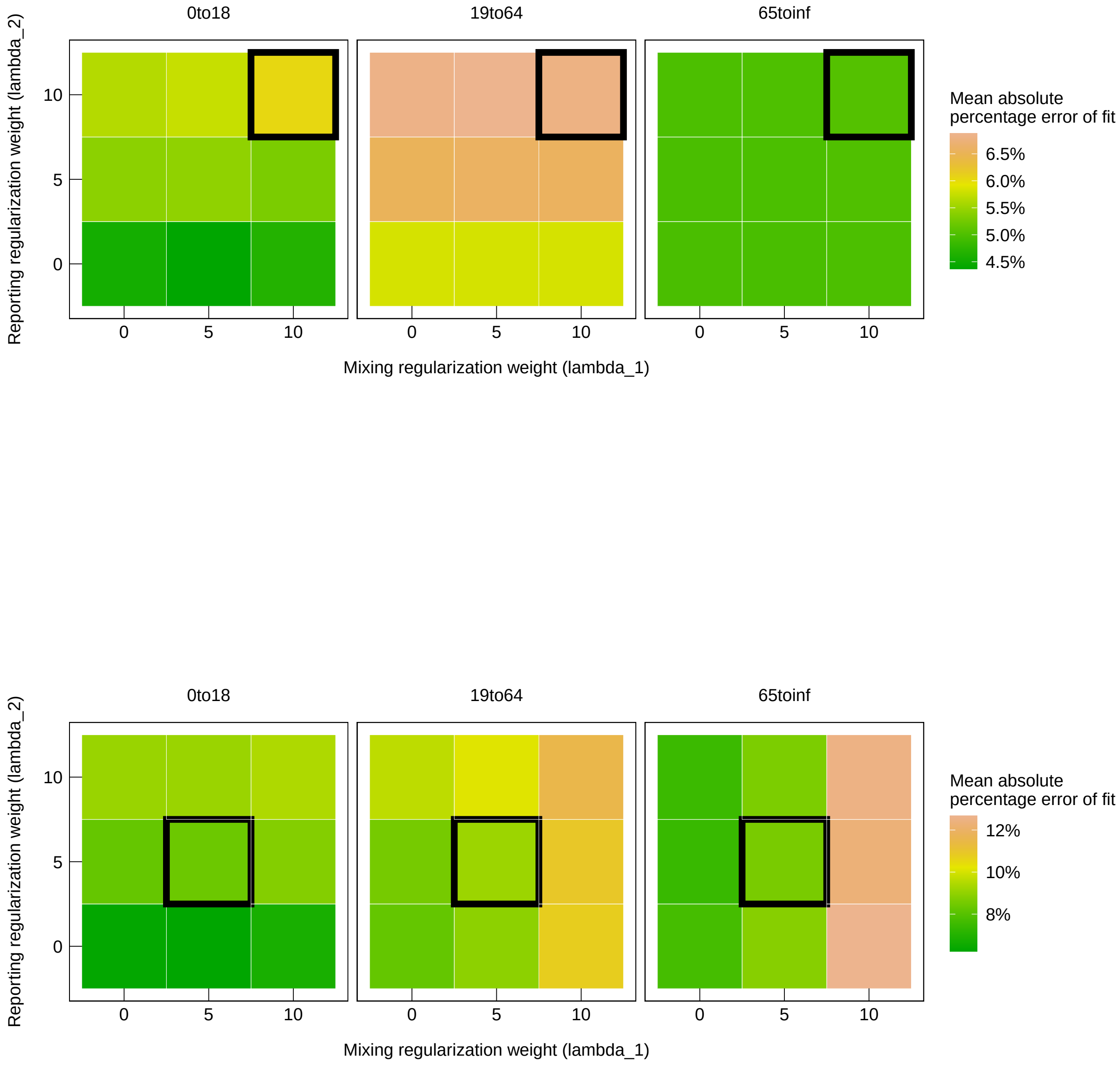

Figure 2: Median values of the mean absolute percentage achieved by the fitted model across all bootstrap datasets as the regularization weights vary, for both the first wave (top panel) and second wave (bottom panel). The selected weights were chosen to be as large as possible, whilst retaining a good fit to the data (low mean absolute percentage error).

References

[1] NPHO of Greece. COVID-19 Vaccinations in Greece [Unpublished raw data]. Greek National Public Health Organization; 2021.

[2] Nyberg T, Ferguson NM, Nash SG, Webster HH, Flaxman S, Andrews N, et al. Comparative analysis of the risks of hospitalisation and death associated with SARS-CoV-2 omicron (B. 1.1. 529) and delta (B. 1.617. 2) variants in England: a cohort study. The Lancet. 2022;399(10332):1303-12.

[3] Braeye T, Catteau L, Brondeel R, van Loenhout JAF, Proesmans K, Cornelissen L, et al. Vaccine effectiveness against onward transmission of SARS-CoV2-infection by variant of concern and time since vaccination, Belgian contact tracing, 2021. Vaccine. 2022;40(22):3027-37. Available from: https://www.sciencedirect.com/science/article/pii/S0264410X22004418.

[4] Ma Q, Liu J, Liu Q, Kang L, Liu R, Jing W, et al. Global percentage of asymptomatic SARS-CoV-2 infections among the tested population and individuals with confirmed COVID-19 diagnosis: a systematic review and meta-analysis. JAMA network open. 2021;4(12):e2137257-7.

[5] Funk T, Innocenti F, Dias JG, Nerlander L, Melillo T, Gauci C, et al. Age-specific associations between underlying health conditions and hospitalisation, death and in-hospital death among confirmed COVID-19 cases: a multi-country study based on surveillance data, June to December 2020. Eurosurveillance. 2022;27(35):2100883.

[6] NPHO of Greece. Self-Testing Figures and Cases Confirmed Positive [Unpublished raw data]. Greek National Public Health Organization; 2021.

[7] Hellenic Statistical Authority. 2011 Population-Housing Census; 2011. https://www.statistics.gr/el/statistics/-/publication/SAM03/2011.

[8] Byrne AW, McEvoy D, Collins AB, Hunt K, Casey M, Barber A, et al. Inferred duration of infectious period of SARS-CoV-2: rapid scoping review and analysis of available evidence for asymptomatic and symptomatic COVID-19 cases. BMJ open. 2020;10(8):e039856.

[9] NPHO of Greece. Length of Hospital Stays for COVID-19 Patients in Greece [Unpublished raw data]. Greek National Public Health Organization; 2021.

[10] NPHO of Greece. Hospitalizations and Deaths for COVID-19 Patients in Greece [Unpublished raw data]. Greek National Public Health Organization; 2021.

Outcome	Model Estimate	Data
Hospitalizations	$\Delta_{ISS_{av} \to H_{av}}(t)$	$\overline{\Delta H}_{av}(t)$
Deaths	$\sum_{v \in \{0,1\}} \Delta_{H_{av} \to D_{av}}(t)$	$\overline{\Delta D}_{a}(t)$
Total Cases	$\sum_{v \in \{0,1\}} \sum_{X \in \mathcal{X}_{av}} P_X^{self}(t) + P_X^{reg}(t)$	$\overline{P}_a^{\text{tot}}(t)$
Cases through Self-Test Program	$\sum_{v \in \{0,1\}} \sum_{X \in \mathcal{X}_{av}} P_X^{self}(t) + \gamma(t) P_X^{reg}(t)$	$\overline{P}_a^{\text{conf}}(t)$

Table 1: Model estimates and observed data used for model fitting.

Parameter	Count (Wave 1)	Count (Wave 2)	Notes
$IAM_{av}(0)$	3	6	initial prevalence $< 0.3\%$
$ISM_{av}(0)$	3	6	initial prevalence $< 0.3\%$
$ISS_{av}(0)$	3	6	initial prevalence $< 0.3\%$
$\beta_{ab}(t)$	36	42	constant over 4 week intervals
p_{av}^{severe}	6	6	intervals from[5]
p_a^{die}	3	3	intervals from[10]
$\gamma_a(t)$	18	21	constant over 6 week intervals
Total	72	90	

Table 2: Number of parameters learned by the model in each of the two waves. We use a sparsity term in the objective to prevent overfitting.

[11] NPHO of Greece. Daily Covid-19 Cases. Greek National Public Health Organization; 2021. https://eody.gov.gr/category/covid-19/.

[12] Arroyo-Marioli F, Bullano F, Kucinskas S, Rondón-Moreno C. Tracking R of COVID-19: A new real-time estimation using the Kalman filter. PloS one. 2021;16(1):e0244474.

[13] Bastani H GVea Drakopoulos K. Efficient and targeted COVID-19 border testing via reinforcement learning. Nature. 2021;599:108–113.

[14] Newville M, Otten R, Nelson A, Ingargiola A, Stensitzki T, Allan D, et al.. lmfit/lmfit-py: 1.0.3. Zenodo; 2021. Available from: https://doi.org/10.5281/zenodo.5570790.

[15] Reuther A, Kepner J, Byun C, Samsi S, Arcand W, Bestor D, et al. Interactive supercomputing on 40,000 cores for machine learning and data analysis. In: 2018 IEEE High Performance extreme Computing Conference (HPEC). IEEE; 2018. p. 1-6.
